# Analyzing potential shifts in life expectancy: The role of baseline mortality in addition to relative changes in death rates

**DOI:** 10.1073/pnas.2404342121

**Published:** 2024-06-03

**Authors:** Sergey Timonin, Caleb C. Cooley

**Affiliations:** ^a^School of Demography, College of Arts and Social Sciences, The Australian National University, Canberra, ACT 2601, Australia; ^b^Department of Sociology, Washington State University, Pullman, WA 99201

To further explore a significant stagnation in life expectancy in the United States, Abrams et al. proposed a counterfactual approach in which the death rates observed in 2000 to 2009 are extrapolated into 2010 to 2019 (1). Their results show that sustained improvements in mortality at older ages, rather than at working ages, may have contributed more to the increase in life expectancy in the United States. Polizzi and Dowd suggest another counterfactual scenario: What would be the contribution of ages 25 to 64 versus 65+ to the life expectancy increase in the United States if relative changes in death rates were similar to those observed in other high-income countries in 2010 to 2019? (2) Conversely, their analysis highlights the vital role of midlife mortality to the changes in life expectancy.

Period life expectancy often serves as a key summary indicator of a population’s health, frequently used to direct health policies and research aimed at understanding and forecasting mortality trends. This aggregated measure is often interpreted as a reflection of mortality conditions within a given year, with year-to-year dynamics dependent on relative changes in age-specific death rates. Additionally, life expectancy alterations hinge on the underlying age-specific mortality pattern, with populations with a less dispersed age-at-death distribution experiencing smaller gains in life expectancy (3).

Building upon this discourse, this letter introduces another (opposite) counterfactual inquiry: What would have been the life expectancy trajectory from 2010 to 2019 in other countries had they mirrored the USA’s mortality change pace during the same timeframe? Our analysis aims to illustrate the potential shifts in life expectancy in diverse mortality contexts, assuming identical changes in age-specific death rates.

**Fig. 1.**
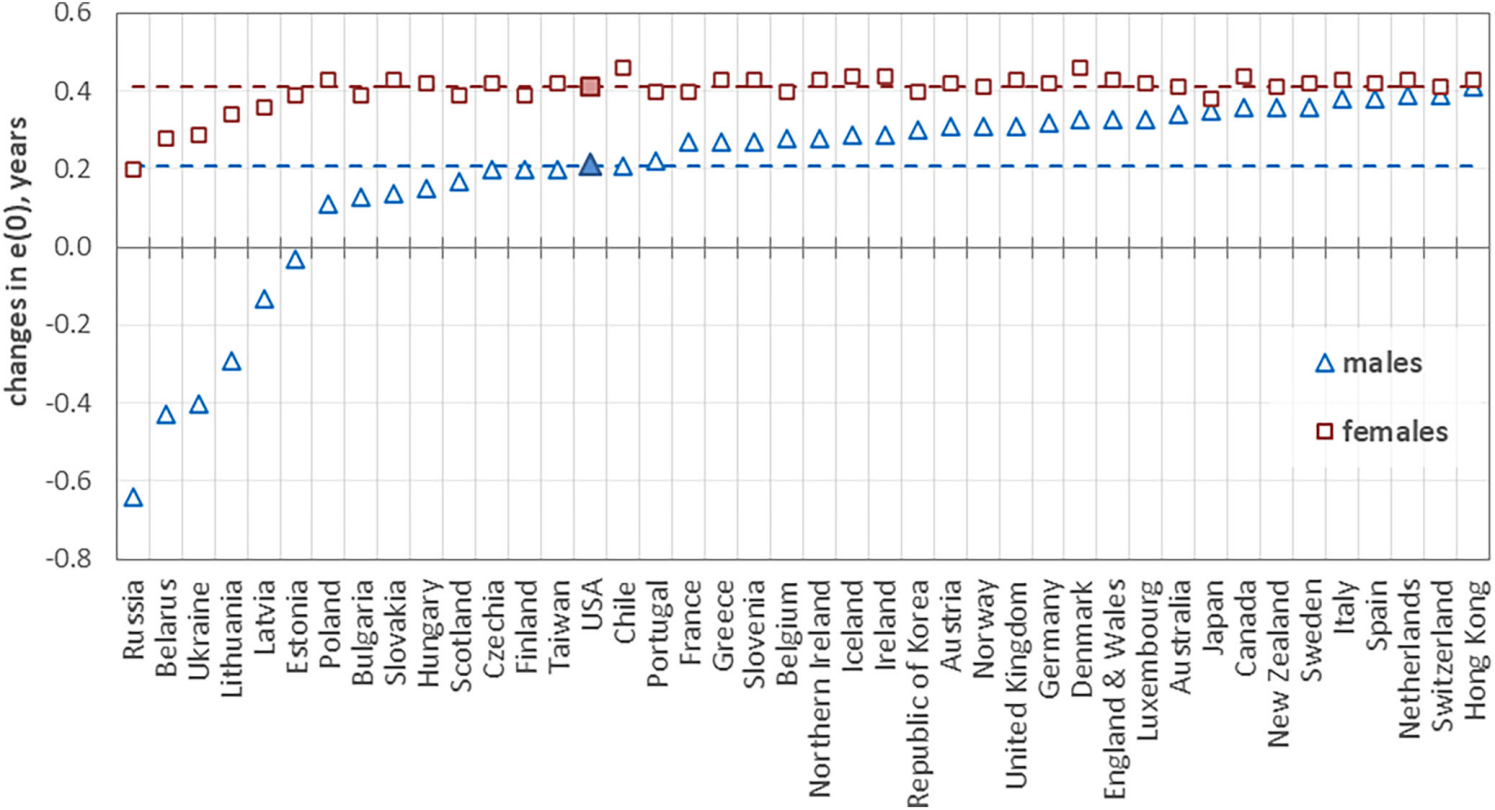
Contrafactual changes in life expectancy at birth (e(0)), by sex, in years. Note: Countries are ranked by the size of the change in life expectancy in males (from negative to positive). The horizontal dashed lines show the actual increase in life expectancy in the United States in 2010 to 2019: blue line for males (0.21 y) and red line for females (0.41 y).

Utilizing mortality data from the Human Mortality Database (4) for 42 countries, including the United States, this analysis leverages the divergent pattern of USA’s mortality changes from 2010 to 2019 as a standard for counterfactual estimations. The results, illustrated in Fig. 1, show marked disparities in potential life expectancy changes across countries, with a *decrease* of 0.64 y in male life expectancy in Russia, a stark contrast to a 0.41-y *increase* in Hong Kong, highlighting the varied outcomes of applying identical relative changes in age-specific death rates to populations with differing baseline mortality schedules.

**Fig. 2.**
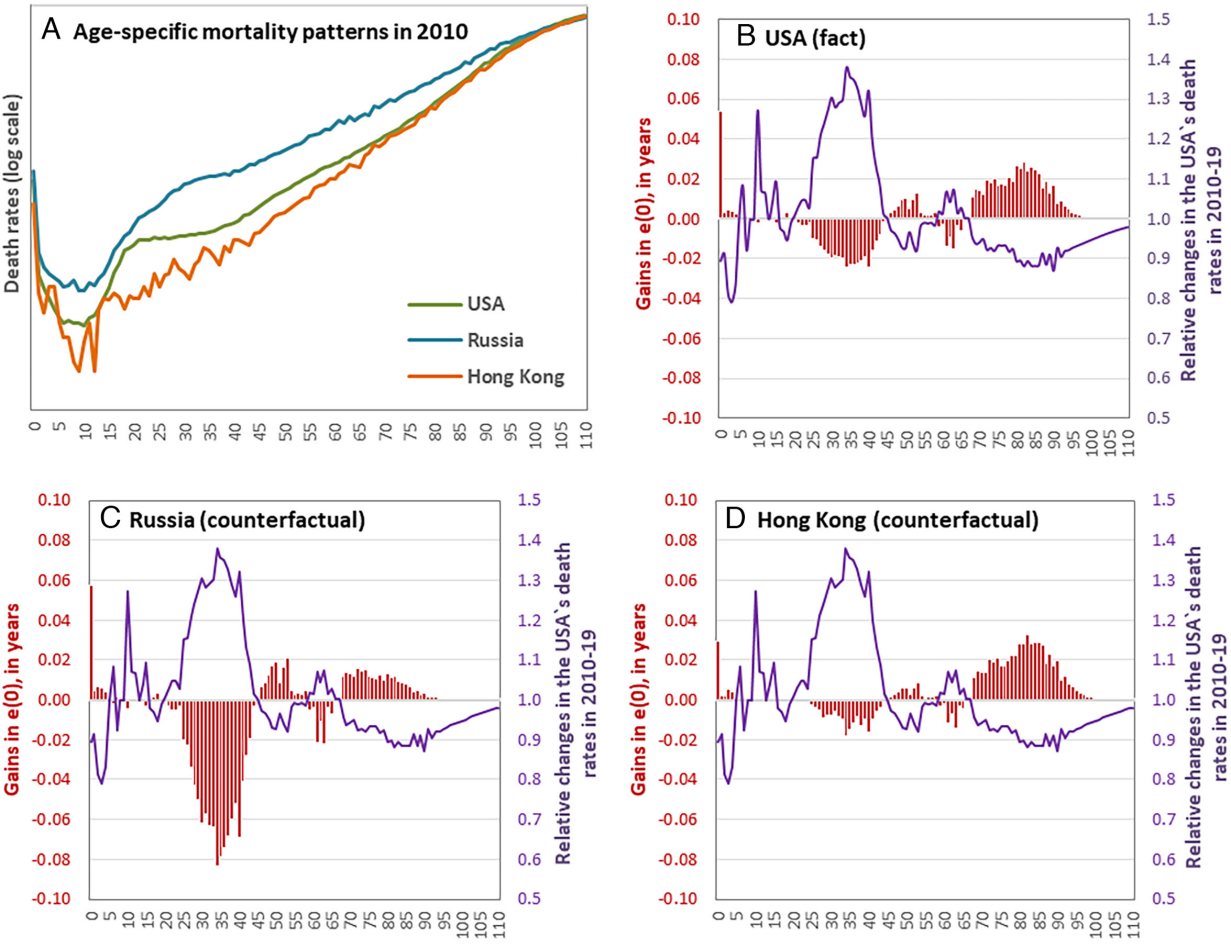
Baseline mortality in the United States, Russia and Hong Kong in 2010 (*A*); relative changes in the age-specific death rates in the United States in 2010 to 2019 and the corresponding age contributions to the changes in life expectancy at birth (*B*–*D*), males.

Fig. 2 demonstrates the contribution of various ages to the counterfactual changes in life expectancy using male populations of Russia and Hong Kong as examples (5). Considering the varied baseline mortality across these populations (Fig. 2*A*), an increase in death rates among individuals aged 20 to 45 could result in a modest decrease in life expectancy by 0.18 y in Hong Kong but a 1-y drop in Russia, with 0.35-y change in the United States. Conversely, improvements in death rates among individuals aged 70 and older could lead to an increase in life expectancy by 0.52 y in Hong Kong, less than half of that in Russia (0.21 y), and 0.45 y in the United States.

In conclusion, using the mortality data for the United States and other countries from the Human Mortality Database, this demographic exercise highlights the importance of considering the interaction between relative changes in death rates and the baseline mortality patterns when utilizing counterfactual approaches to investigate life expectancy changes.
